# Gene Promoter-Methylation Signature as Biomarker to Predict Cisplatin-Radiotherapy Sensitivity in Locally Advanced Cervical Cancer

**DOI:** 10.3389/fonc.2022.773438

**Published:** 2022-03-03

**Authors:** Carlos Contreras-Romero, Eloy-Andrés Pérez-Yépez, Antonio Daniel Martinez-Gutierrez, Alma Campos-Parra, Alejandro Zentella-Dehesa, Nadia Jacobo-Herrera, César López-Camarillo, Guillermo Corredor-Alonso, Jaime Martínez-Coronel, Mauricio Rodríguez-Dorantes, David Cantu de León, Carlos Pérez-Plasencia

**Affiliations:** ^1^Laboratorio de Genómica, Insituto Nacional de Cancerología, Ciudad de México, Mexico; ^2^Cátedra CONACYT, Dirección de cátedras, Consejo Nacional de Ciencia y Tecnología (CONACYT), Mexico City, Mexico; ^3^Programa Institucional de Cáncer de Mama, Dpto Medicina Genómica y Toxicología Ambiental, IIB, Universidad Nacional Autónoma de México (UNAM), Mexico City, Mexico; ^4^Unidad de Bioquímica, Instituto Nacional de Ciencias Médicas y Nutrición Salvador Zubirán (INCMNSZ), Ciudad de México, Mexico; ^5^Posgrado en Ciencias Genómicas, Universidad Autónoma de la Ciudad de México (UACM), Mexico City, Mexico; ^6^Laboratorio de Patología, Hospital General de Zona #92, Ciudad Acuña, Mexico; ^7^Laboratorio de Oncogenómica, Instituto Nacional de Medicina Genómica, Mexico City, Mexico; ^8^Laboratorio de Genómica, Unidad de Biomedicina, FES-Iztacala, UNAM, Tlalnepantla, Mexico

**Keywords:** gene promoter methylation, chemoradioresistance, cervical cancer, biomarkers, Cisplatin-Radiotherapy sensitivity

## Abstract

Despite efforts to promote health policies focused on screening and early detection, cervical cancer continues to be one of the leading causes of mortality in women; in 2020, estimated 30,000 deaths in Latin America were reported for this type of tumor. While the therapies used to treat cervical cancer have excellent results in tumors identified in early stages, those women who are diagnosed in locally advanced and advanced stages show survival rates at 5 years of <50%. Molecular patterns associated with clinical response have been studied in patients who present resistance to treatment; none of them have reached clinical practice. It is therefore necessary to continue analyzing molecular patterns that allow us to identify patients at risk of developing resistance to conventional therapy. In this study, we analyzed the global methylation profile of 22 patients diagnosed with locally advanced cervical cancer and validated the genomic results in an independent cohort of 70 patients. We showed that BRD9 promoter region methylation and CTU1 demethylation were associated with a higher overall survival (p = 0.06) and progression-free survival (p = 0.0001), whereas DOCK8 demethylation was associated with therapy-resistant patients and a lower overall survival and progression-free survival (p = 0.025 and p = 0.0001, respectively). Our results suggest that methylation of promoter regions in specific genes may provide molecular markers associated with response to treatment in cancer; further investigation is needed.

## 1 Introduction

Cervical cancer (CC) is the fourth most common type of cancer in women worldwide (1). In developing countries, mainly in Latin America, about 30,000 deaths per year are caused by this disease (2). The high mortality rates are due to the fact that 50% of patients are diagnosed in locally advanced cervical cancer stages (LACC); the overall survival (OS) rate to 5 years is approximately 60% (1) and a recurrence rate from 15% to 40% (3). Conventional treatment for LACC patients consists of concomitant chemoradiotherapy. Unfortunately, treatment resistance is observed in approximately 30% of patients (4).

Treatment resistance involves several molecular alterations such as genetic mutations, dysregulated microRNAs, dysregulated long noncoding RNAs expression profiles, and epigenetic modifications (5–9). Several reports described that the aberrant DNA methylation that involves hypo- or hypermethylation is also associated with tumor progression and therapy resistance (10, 11). For example, the hypermethylation of PTEN, MYOD1, RASSF1A, APC1A, PTGS2, and VIM genes, which are associated with OS of CC patients, covered all stages (12–16). Nevertheless, the expanding knowledge about methylation profiles in patients with LACC is pertinent and is focused on the treatment resistance in these particular patients.

The goal of this study was to obtain the global methylation pattern of tumor biopsies from 92 LACC patients treated with chemoradiotherapy to identify the methylation status of specific gene promoters with predictive potential to the cisplatin-radiotherapy response. For this purpose, we analyzed the methylation profile in 22 patients and found global changes in methylation patterns in 7,957 gene promoter regions that distinguish responsive and resistant LACC patients to chemoradiation. Next, by means of bioinformatics tools, we selected promoter sequences with a CpG density higher than 60%; these regions corresponded to the promoters of the bromodomain containing 9 (BRD9), dedicator of cytokinesis 8 (DOCK8), and cytosolic thiouridylase subunit 1 (CTU1) genes. Then, promoter regions were experimentally validated by methylation-specific PCR (MSP) in an independent cohort of 70 LACC patients. Strikingly, we found a correlation between BRD9 promoter region methylation and CTU1 demethylation with complete response to chemoradiotherapy in addition to higher overall survival (OS) (p = 0.06) and progression-free survival (PFS) (p = 0.0001). Moreover, demethylation of DOCK8 promoter region was associated with patients who developed treatment resistance and lower OS and PFS (p = 0.025 and p = 0.0001, respectively). These data point to the methylation status of BRD9 CTU1 and DOCK8 as potential biomarkers for predicting survival and response to chemoradiotherapy in LACC patients.

## 2 Material and Methods

### 2.1 Tissue Samples

This study was approved by the Central Ethics and Scientific Committee at the National Cancer Institute in Mexico City (INCan) (015/012/ICI, CEI/961/15) and has been conducted in agreement with the ethical standards as laid down in the 1964 Declaration of Helsinki and its later amendments. A total of 92 biopsies from patients with LACC cancer were obtained. Tumor samples were collected from 2014 to 2018 by the Pathology Department, INCan, Mexico City. After confirmed diagnosis, all patients received concurrent chemoradiotherapy using cisplatin [weekly cis-diamminedichloroplatinum (II) at a dose of 40 mg/m^2^] for a total of five or six cycles and radiation (external radiation and intracavitary brachytherapy, for a total dose of 64–66 Gy over 67 days) (17). The patients’ therapy response was assessed according to RECIST criteria defined as follows: the disappearance of all target lesions was assigned as complete response (CR); meanwhile, patients with partial response, progressive disease, or stable disease were considered as therapy resistant (TR). The biopsies were divided into two cohorts: the first with 22 patients (12 CR and 10 TR) used as a discovery cohort to generate a microarray specific for CpG islands Array-Based Profiling of Reference-Independent Methylation Status (aPRIMES) (18); the second cohort, with 70 biopsies (40 CR and 30 TR), used for molecular data validation. The patient eligibility criteria consisted of (a) confirmed pathological diagnosis of CC stages from II-B to IV-B (LACC), (b) biopsies with a pathology report confirming more than 80% tumorous cells, (c) age range of 29–65 years, (d) high-quality DNA and RNA samples, (e) no other comorbidity, (f) no previous oncological treatment, and (g) patients able to receive the standard therapy based on concurrent chemotherapy and radiotherapy.

### 2.2 Nucleic Acid Extraction

The DNA extraction from the 92 biopsies was performed as follows: 20 mg of fresh tissue was placed in a Fisherbrand Bead Mill homogenizer, and 2 ml soft tissue homogenizing Mix Tube was preloaded with lysis buffer [10 mM Tris–HCl, 2 mM ethylenediaminetetraacetic acid (EDTA), 1% sodium dodecyl sulfate (SDS)]. The tissue was homogenized using the MagNA Lyser instrument at 6,000 rpm for 1 min. To purify the genomic DNA, the QIAamp DNA Blood Kit (Qiagen, CA, USA) was used according to the manufacturer’s protocol. Finally, the purified DNA was stored at −20°C.

### 2.3 Microarray Differential Methylation Analysis (aPRIMES)

We employed the 3x720K CpG Island Plus RefSeq Promoter Arrays (Roche, Penzberg, Germany). These arrays cover the annotated CpG islands and the promoters of the RefSeq genes derived from the UCSC RefFlat files (Hg 38). Then, the hybridization probes were synthesized by aPRIMES assay. Briefly, genomic DNAs were digest by *Mse*I, and the fragments obtained were subjected to linker-mediated PCR as described by Klein and coworkers (19); later, through enzymatic digestion by methylated-sensitive and methylated-specific enzymes, we obtained a methylated and unmethylated fraction of DNA, which were labeled with Cy5 and Cy3 fluorophores, respectively, and competitive hybridizing in a Human DNA Methylation 3x720K CpG Island Plus (Roche) as shown in **Supplementary Figure 1**. Then, arrays were scanned in an MS200 scanner (Roche). Finally, the alignment of the images and the extraction of data were carried out using the software DEVA Project Manager—1.2.1 (Roche). Next, for each region in the array, we obtained a continuous numerical ratio that represents if a region is hyper- or hypomethylated; we termed this ratio as bi-weight (BW) and is represented with the following formula:


$$BW=\frac{Log2(Cy3)}{Log2(Cy5)}$$

Finally, to determine the significative methylated regions between responsive and resistant tumors, we calculated the Student’s t-test for each region between both groups and considered as statistical significance those methylated regions with a p < 0.01. Then, selected regions were ranked in ascending and descending orders accordingly to the difference of the means for both groups. All statistical analysis were executed in R environment.

### 2.4 Pathway Analysis

Differentially methylated regions were analyzed by Pathway enrichment analysis by using Webgestalt (20) and ReactomePA (21); we only considered pathways with a p < 0.05 as subject of regulation.

### 2.5 CpG Island Density Determination

We obtained from the Genome Browser database (22) a sequence of 2,000 bp (1,000 bp downstream; transcription start site, 1,000 bp upstream) that included the promoter region from each analyzed gene. These sequences were analyzed using MethPrimer web tool from Urogene to determine the CpG density (23).

### 2.6 Methylation-Specific PCR Assay

To determine the methylation status of selected genes (BRD9, CTU1, and DOCK8), genomic DNA from each sample was modified using Methylation-Direct EZ DNA Kit (ZYMO, CA, USA). DNA bisulfite treatment changed unmethylated cytosines to uracil, but the methylated bases remained as cytosines. Then, two PCR reactions were performed per sample using specific primers to determine the methylated (M) or unmethylated (U) DNA status. The list of primers and its characteristics are shown in **Supplementary Table S1**. The product of each reaction was analyzed in agarose gels and resolved in Minigel OWLTM Easy CastTM B2 system (Thermo Scientific, MA, USA). Later, the gel was stained with ethidium bromide and photo-documented using a Gel Doc EZ Imager transilluminator (Bio Rad, CA, USA).

### 2.7 Statistical Analysis

Chi-squared tests were employed to determine the differences in the distribution of the methylation status of the genes and the clinicopathological characteristics, considering p < 0.05 as statistically significant.

### 2.8 Survival Analysis

Kaplan–Meier plotter was calculated using the survival package in R, where the significance testing was assessed using the log-rank test. Significance was considered as p < 0.05.

## 3 Results

### 3.1 Clinicopathological Characteristics of Patients

This study was approved by the Central Ethics and Scientific Committee at the National Cancer Institute in Mexico City (approval number 015/01271B/CEI/961/15). The 92 patients who were enrolled accepted and signed the informed consent. All patients received treatment based on cisplatin and radiotherapy as mentioned in *Material and Methods*. The median age was 48 years. Patients were classified following the last version of International Federation of Gynecology and Obstetrics (FIGO) staging criteria as II (51.8%), III (37%), and IV (11.2%) stages. According to the FIGO’s guidelines, 52 patients (56.52%) showed complete response (CR) to therapy; meanwhile, 40 (43.48%) exhibited therapy resistance (TR). The HPV-genotype of all patients was determined by nested PCR (24). **Table 1** shows the clinicopathological characteristics; a supplementary table that compiles all clinical data is available as **Supplementary Table 2**.

**Table 1:** Clinicopathological characteristics of LACC patients.

Clinicopathological characteristics N= 92 (%)		
Histological type		
Epidermoid	83 (90.27%)	
Adenocarcinoma	9 (9.73%)	
Clinical stage (FIGO)		
II	55 (59.78%)	Stage II: TR^a^=25.5% CR^b^=74.5%
III	27 (29.34%)	Stage III: TR= 57.1% CR=42.9
IV	10 (10.88%)	Stage IV: TR=100%
Age (29–63) years		
29-39	18 (19.56%)	
40-49	25 (27.17%)	
50-61	27 (29.34%)	
Older than 61	22 (23.93%)	
Tumor size		
≥5 cm	37	
< 5cm	55	
	Median= 4.01	
HPV genotype		
16	51 (55.43%)	
18	22 (23.91%)	
52	8 (8.69%)	
58	5 (5.46%)	
6	3 (3.26%)	
59	2 (2.17%)	
33	1 (1.08%)	

a TR: percentage of patients who developed therapy resistance.

b CR: percentage of patients who had complete response to conventional treatment.

### 3.2 Global Analysis of DNA Methylation in Locally Advanced Cervical Cancer Tumors

**Figure 1:**
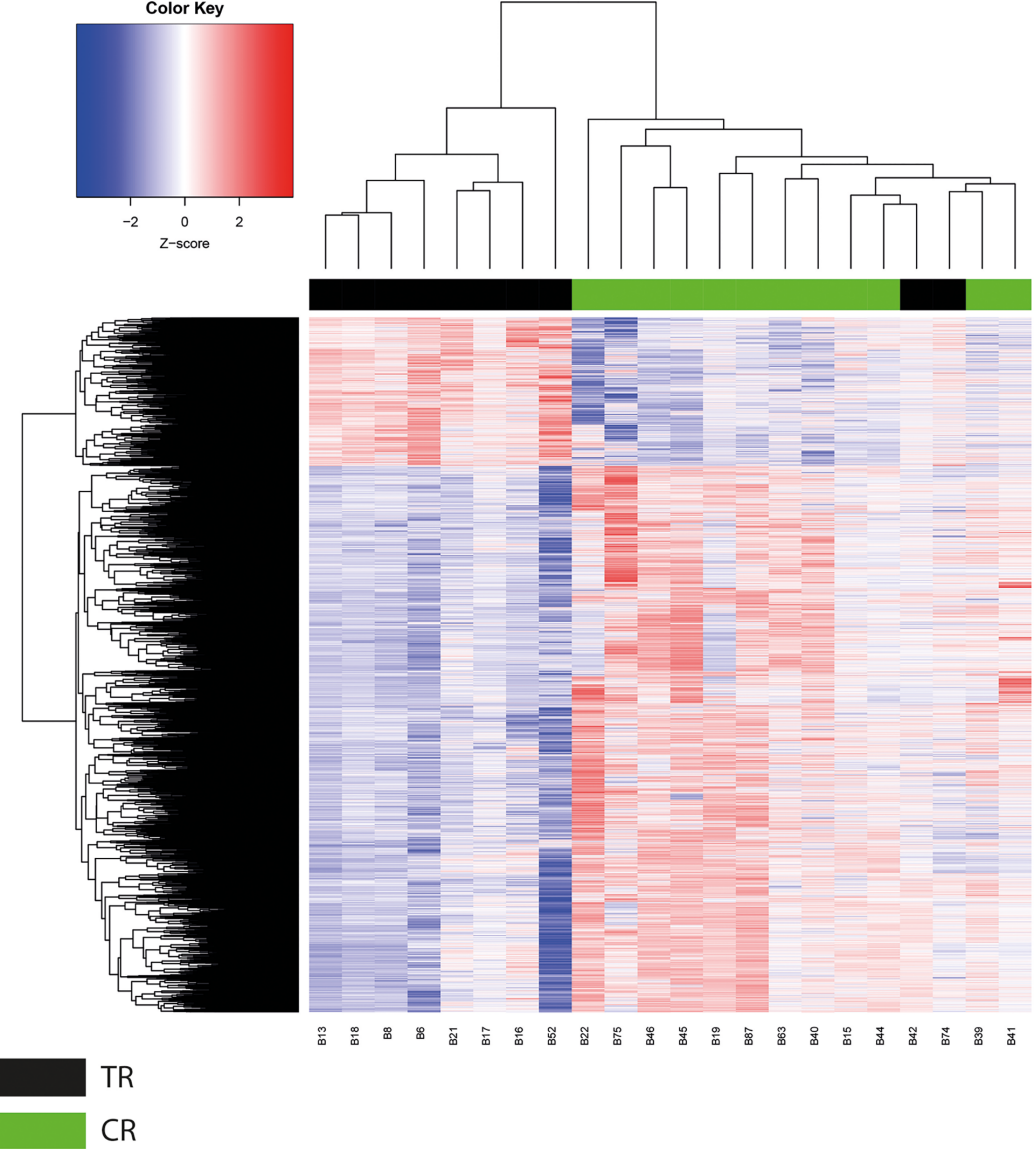
Global methylation analysis. Unsupervised clustering analysis of 16,538 CpG regions differentially methylated between therapy resistance (TR) and complete response (CR) tumors. Red color regions represent high levels of methylation (Z score from 0 to 2), and blue color regions represent low methylation status (Z score from 0 to −2).

The determination of DNA methylation patterns has been proposed as a prognosis predictor in several types of cancer (25, 26). In this study, we employed aPRIMES arrays to obtain the genome-wide DNA methylation patterns on both groups, namely, responsive (CR) and therapy-resistant (TR) **LACC** patients. Next, to establish the differentially methylated regions (DMRs) on CR and TR groups, we compared the bi-weight ratio values from each analyzed region, using a Student’s t-test. The results showed a methylated DNA profile between the two groups composed of 16,538 DMRs that corresponded to 7,957 unique regions, where 2,833 of them were hypermethylated, 5,881 were hypomethylated, and 757 regions had both hyper and hypomethylated DMRs regions (p < 0.05) (**Figure 1**). As expected, a global hypomethylation pattern across the genome was observed, where the distribution of these DMRs varied to a considerable extent depending on the chromosome (**Supplementary Figure S2**). We noticed that chromosomes 1 and 19 had the higher number of gene promoters with DMRs, 859 and 753, respectively (**Supplementary Figures S2**, **S3**). Next, we observed that clustering of these DMRs using the Euclidean distance algorithm could distinguish the TR (black bar) from CR LACC patients (green bar) (**Figure 1**). Since the bi-weight ratios are continuous variables, we transformed them to a Z-score to visualize the DMRs in a heatmap, which clearly shows a DNA methylation profile that includes 3,533 DMRs hypermethylated and 13,005 DRMs hypomethylated (**Figure 1**).

### 3.3 Gene Pathway Analysis

**Figure 2:**
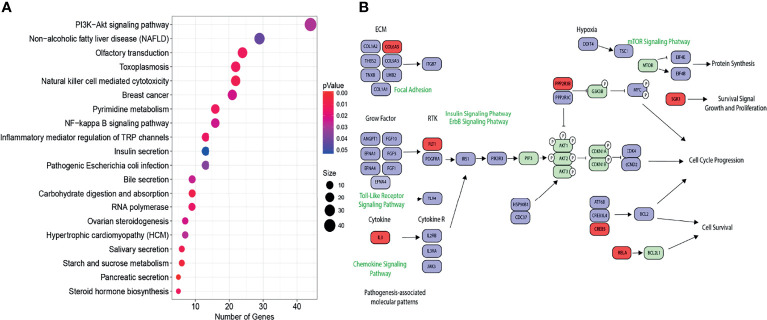
**(A)** KEGG analysis. Signaling pathways with a p < 0.05 as subject of regulation by the methylated/unmethylated genes. The dot size is according to number of the related genes for each pathway. The color of the dots is represented by the range of colors from blue to red depending of the p-value. **(B)** The PI3K-AKT pathway, where blue represents hypomethylated genes and red represents hypermethylated genes.

Furthermore, we were interested in evaluating the impact of the methylation profile in biological pathways. The gene set enrichment analysis using the Kyoto Encyclopedia of Genes and Genomes (KEGG) database revealed that multiple key carcinogenic pathways such as the PI3K-AKT signaling pathway, nuclear factor (NF)-kappa B pathway, RNA polymerase, and pathways associated with breast cancer were dysregulated as a consequence of differentially methylation profile (**Figure 2**). As the PI3K-Akt pathway was the most enriched, we focused on analyzing it in more detail. As shown in **Figure 2**, multiple key genes in this pathway were hypomethylated, including the insulin receptor substrate-1 (IRS-1) and oncogene JAK3. In contrast, genes such as RELA that inhibit the tumor growing were hypermethylated.

### 3.4 The Methylation Status of BRD9, CTU1, and DOCK8 Gene Promoter Regions Is Associated With Clinical Outcomes of LACC Patients

**Figure 3:**
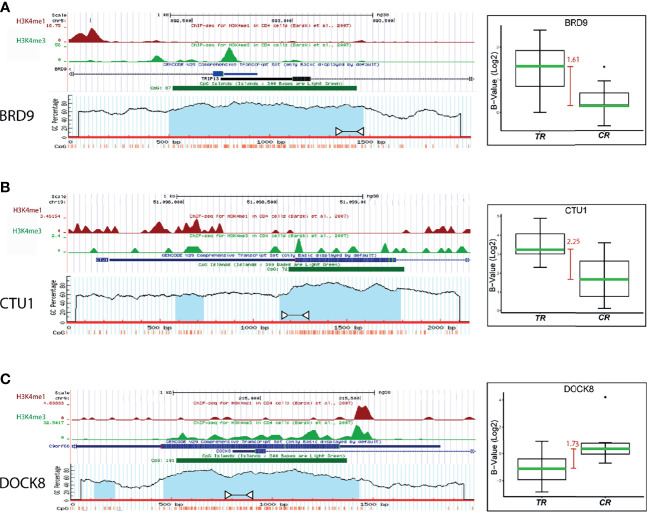
Analysis of the promoter regions of **(A)** BRD9, **(B)** CTU1, and **(C)** DOCK8 genes. Blue bar represents the promoter region of each gene, the green bar points the CpG island location, and the blue shadow color indicates the CpG density of the island. White arrows indicate the amplification region for the MSP validation. The boxplot shows the median difference (MD, red bars) between TR and CR samples methylation levels from each promoter region.

To select methylated genes as potential biomarkers of response to chemoradiation, we further narrowed the methylation profile by considering only those DMRs that showed hyper- or hypomethylation status for further analysis. The results showed 4,463 DMRs with these methylation patterns that correspond to 1,439 unique genes. Then, median bi-weight values of methylation from each gene in TR and CR tumors were compared to calculate the median difference (MD). Promoter regions with an MD upper than 1.4 times, corresponding to the 13 genes enlisted in **Table 2**, were chosen for further analysis. The promoter sequence of the selected genes was analyzed as mentioned in *Section CpG Island Density Determination*, and the three genes with highest CpG density were selected for validation. A CpG island is defined as a DNA region highest than 500 bp that contains 50% or more of CG dinucleotides (27). The promoter region of BRD9, CTU1, and DOCK8 genes showed 88%, 63%, and 90% of CpG density, respectively (**Figure 3**). Additionally, the MD for these promoter regions was 1.61, 2.25, and 1.73 for BRD9, CTU1, and DOCK8, respectively (**Figure 3**, boxplots). Interestingly, H3K4me3 mark (chromatin compaction mark) was found near to these promoter regions (**Figure 3**).

**Table 2:** Genes with the highest differential MD value.

Gen Name	MD
1. KIAA1539	2.4407
2. DCTPP1	2.3529
3. STAG3L3	2.3171
4. CTU1	2.2560
5. SLC17A7	2.2266
6. EPB41L1	1.7668
7. DOCK8	1.7396
8. PRPF40B	1.6712
9. HPS1	1.6231
10. TUBGCP2	1.6421
11. BRD9	1.6130
12. RNASEH2A	1.4513
13. SNX17	1.4315

**Figure 4:**
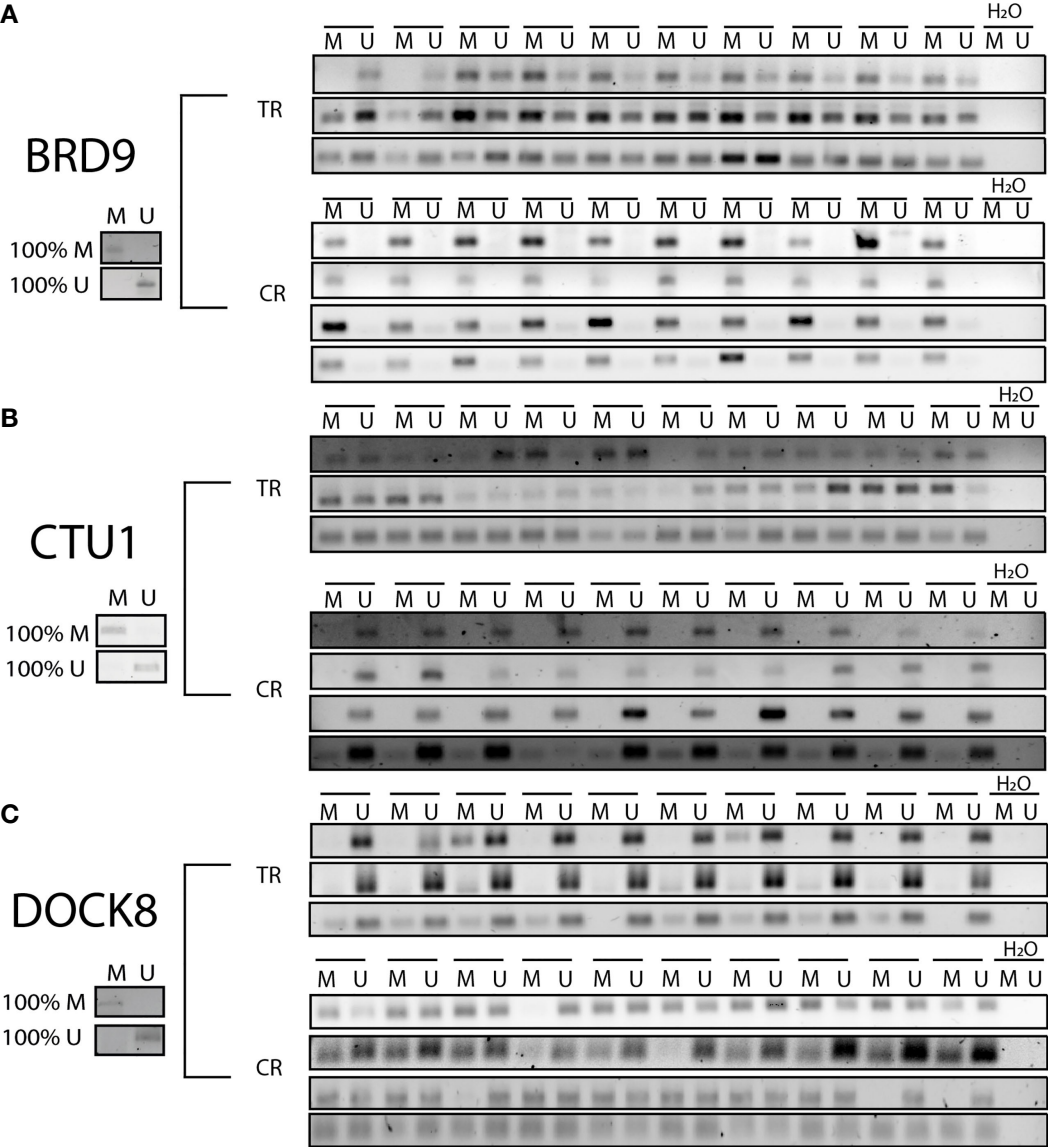
Methylation status of BRD9, CTU1, and DOCK8 promoter regions. Products from methylation-specific PCR (MSP) assay were resolved in agarose gels. A representative gel to each evaluated gene is shows in the figure. Twenty biopsies TR and 20 CR were processed to verify the methylation (M), unmethylation (U), or hemimethylation (HM) status of promoter region to **(A)** BRD9, **(B)** CTU1, and **(C)** DOCK 8. As control for each PCR reaction, 100% methylated (100% M) and 100% unmethylated (100% U) DNA were used.

To validate the methylation levels of BRD9, DOCK8, and CTU1 promoter regions as therapy response biomarkers, an MSP assay was performed. Bisulfite-treated DNA from 30 TR and 40 CR tumors samples were used to analyze methylated status. The results showed that the BRD9 promoter region was methylated in all CR tumor samples (40 CR tumor samples, 100% of cases), whereas it was hemimethylated in 25 TR tumor samples and unmethylated in 5 TR tumor samples (83% and 17% of cases, respectively) (**Figure 4A**). Instead, the promoter region of CTU1 gene was detected to be unmethylated in all CR tumor samples (40 CR tumor samples, 100% of cases), while in 27 TR tumors samples, it was hemimethylated; in 2 TR tumor samples, it was unmethylated and only in 1 TR tumor sample that it was methylated (90%, 6.6%, and 3.4% of cases, respectively) (**Figure 4B**). On the other hand, the DOCK8 promoter region was unmethylated in 29 TR tumors samples (97% of cases), whereas in 31 CR tumors samples, it was hemimethylated, and in 9 CR tumors samples, it was unmethylated (77.5% and 22.5% of cases, respectively) (**Figure 4C**).

Additionally, a chi-square analysis was performed to compare the methylation status of BRD9, CTU1, and DOCK8 genes with demographic characteristics of LACC patients (**Table 3**). The methylation of the BRD9 gene was associated with tumor stages II and tumor size <5 cm. In contrast, unmethylation of the CTU1 promoter region gene was associate with stages II and with tumor size <5 cm. The unmethylation status of the DOCK8 promoter region showed an association with stages III–IV; however, no significant relationship was found between the methylation status of this promoter with tumor size.

**Table 3:** Chi-Square analysis of gene methylation status and clinical characteristics of patients.

	Methylated	Hemi-methylated	Un-methylated	*p* value
BRD9				
Stage II	32	5	3	**0.00012**
Stage III – IV	8	20	2	
Tumor size < 5cm	30	11	2	**0.04984**
Tumor size ≥ 5cm	9	11	3	
	**Methylated**	**Hemi-methylated**	**Un-methylated**	***p* value**
CTU1				
Stage II	0	8	31	**0.00031**
Stage III – IV	1	20	9	
Tumor size < 5cm	0	14	29	**0.03959**
Tumor size ≥ 5cm	1	16	9	
	**Methylated**	**Hemi-methylated**	**Un-methylated**	***p* value**
DOCK8				
Stage II	0	25	15	** 0.01189**
Stage III – IV	0	8	22	
Tumor size < 5cm	0	20	21	** 0.563584**
Tumor size ≥ 5cm	0	9	16	

The bold values correspond to p value for each correlation chi-square analysis.

### 3.5 A Gene Methylation Signature as Biomarker for Overall Survival and Progression-Free Survival in CC

**Figure 5:**
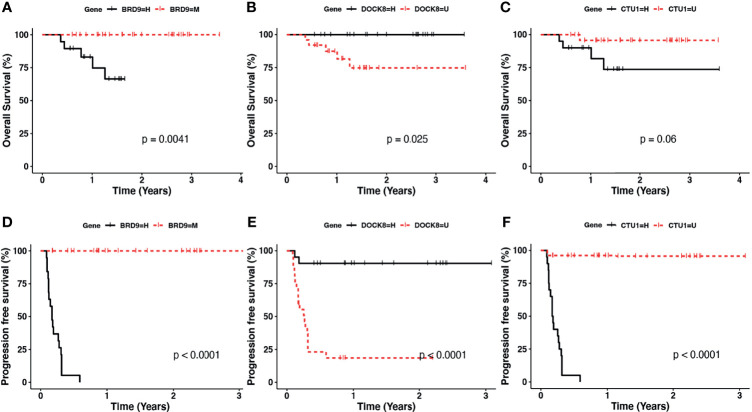
Kaplan–Meier plotter for the methylation status of **(A, D)** BRD9, **(B, E)** DOCK8, and **(C, F)** CTU1 in the overall survival (upper panel) and progression-free survival (lower panel). Black lines correspond to hemimethylated (H) and red line to methylated (M) of analyzed gene promoter region.

Finally, we determined if the methylation status of BRD9, CTU1, and DOCK8 genes could be an OS and the PFS biomarker of LACC patients. The results showed a better OS (p < 0.0041) and PFS (2.28 months in the hemimethylated group, p < 0.0001) in patients with methylation of BRD9 promoter (**Figures 5A, D**). In contrast, worse OS (p < 0.025) and PFS (3.12 months in the unmethylated group p < 0.0001) was observed in patients with the methylation of the DOCK8 promoter (**Figures 5B, E**). Moreover, patients with a unmethylated CTU1 promoter showed a better OS and PFS (1.76 months in the hemimethylated group p < 0.0001) (**Figures 5C, F**). These data highlight that the methylation status of BDR9, CTU1, and DOCK8 have the potential as biomarkers of OS and PFS in LACC patients.

## 4 Discussion

Despite global screening programs, CC remains a health problem in Latin American countries, with an estimated 56,000 new cases and 28,000 cervical cancer deaths (2). Unfortunately, more than 50% of CC patients are diagnosed at locally advanced stages with a 5-year survival rate of 60% (28). Epigenetic processes are crucial in cellular homeostasis, and their dysregulation leads to cancer and progression (29). DNA methylation is a tag for chromatin remodeling factors that have a crucial role in transcription regulation; DNA methylation in promoter regions is considered as a transcriptional repression mark of gene expression (30). The aberrant methylation of genes is a relevant event during carcinogenesis, which could be a diagnostic biomarker of the disease (31, 32). However, few studies are focused on associating the methylation status with the response to treatments in CC patients. Therefore, expanding knowledge about methylation profiles in patients is decisive to build knowledge focused on treatment resistance. In this regard, we aimed to find gene methylation as a biomarker of response to chemoradiotherapy in LACC. Consequently, we performed a global analysis of DNA methylation from chemoradiotherapy-responsive tumor biopsies to establish DNA methylation patterns. Hence, we identified a gene methylation profile that distinguished between responsive patients and resistance to chemoradiotherapy. As mentioned previously, prognostic biomarkers based on chemoradiotherapy-related aberrant DNA methylation are limited. However, a study in head and neck squamous cell carcinoma described a characteristic promoter methylation pattern of ZNF10, TMPRSS12, ERGIC2, and RNF215 genes, which was proposed as a biomarker of response to radiotherapy treatment (33). Another study performed in low-grade gliomas reported a consistent signature in the methylation of MGMT, MLH3, RAD21, and SMC4 promoter region predictive value for response to temozolomide (34). In breast cancer, the hypermethylation of IL15RA gene promoter induced the upregulation of genes involved in adhesion and ECM-interaction pathways correlating with the OS of patients (35). In CC, methylation patterns are used as biomarkers to distinguish between healthy and cancerous tissue (36–39). Besides, methylation of SOCS2 and hTERT promoter region was associated with early-stage tumors (40), while the methylation of the APC1A promoter was related to advanced stages (15). Therefore, methylation profiles could predict cancer stages. Elsewhere, reports indicated the role of gene methylation associated with survival, such as MYOD1 and VIM methylation status associated with more favorable disease-free survival and OS (12, 39, 41). Likewise, our results showed a correlation between the methylation status of BRD9, CTU1, and DOCK8 promoter regions with PFS, OS, and clinicopathological characteristics of LACC patients.

In the present work, we ascertained a signature to predict chemoradiotherapy response in LACC patients. This signature consisted of the methylation of the BRD9 promoter region, the unmethylation of the CTU1 gene, and the unmethylation of the promoter region of DOCK8. Fascinatingly, the methylation of the BRD9 promoter region and unmethylation of CTU1 were related to CR, and the unmethylation status of DOCK8 was related to TR. Furthermore, the methylation signature was validated in an independent cohort, allowing us to propose it as a potential biomarker to predict the response capacity of LACC patients to chemoradiotherapy. In this regard, CpG island methylation from DNA promoter regions leads to the inactivation of genes, some of which are tumor suppressors, whereas the demethylation of those repeats elements induces the gene expression of oncogenes (5).

In our work, we detected the BRD9 gene promoter methylation pattern in CR tumors, suggesting low levels of expression of this gene, which could explain the response rates to chemoradiotherapy. The BRD9 gene encodes a protein that functions as a protein interaction module that recognizes lysine acetylation domains, a key event in the reading of epigenetic marks (40). The overexpression of this gene in lung cancer cells was associated with poor prognosis, and its oncogene role was demonstrated in synovial sarcoma (42).

In this work, we detected CTU1 unmethylated in LACC samples of patients that showed response to chemoradiotherapy and better OS. CTU1 plays a crucial role in the processing of transfer RNA by modifying nucleosides for the precise binding of the anticodon, thus guaranteeing the fidelity of the translation by the ribosome (43). However, its role in CC has not been analyzed yet, but in breast cancer, CTU1 overexpression promotes cell invasion (44). On the other hand, we found that the unmethylation of the promoter region of DOCK8 was detected in TR patients. The role of this gene is unknown in CC. Nevertheless, in a recent work, Biswas and colleagues (45) reported that DOCK8 is a gene that codifies to a nucleotide exchange factor (GEF) that activates the GTPase CdC42, participating in cell migration and invasion. In addition, it was shown acute in myeloid leukemia that its pharmacological inhibition attenuates cell survival (45).

Then, we performed a multi-pathway analysis using the differential methylation pattern established from the comparison between CR and TR tumors. The results showed dysregulated pathways such as PI3K-Akt-mTOR. This pathway regulates multiple cellular and molecular functions like cell cycle progression, cellular growth, and protein synthesis and is altered in various cancer types including CC, which are crucial for tumor initiation, invasion, and metastasis (46). These data suggested that this pathway could be hyperactivated in chemoradiotherapy-resistant LACC patients (**Figure 2**). This is the case of ovarian and breast cancers, where it was shown that hyperactivation of this pathway is related to chemoresistance and drug resistance, respectively (47, 48). There are no studies that corroborate the causality of hyperactivation of the pathway and chemoradiotherapy resistance in CC. Thus, the elucidation of molecular pathways altered by the differential methylation pattern between responsive and resistant cervical tumors remains a perspective to future studies.

In summary, this is the first study to report a molecular signature of promoter methylation of the BDR9, CTU1, and DOCK8 genes, which could distinguish LACC response patients to resistant to chemoradiotherapy. In this regard, we propose them as potential biomarkers of response to chemoradiotherapy in LACC patients. Extending this study to other cohorts and deepening the biological role of these genes are of great interest.

## Data Availability Statement

The datasets presented in this study can be found in online repositories. The names of the repository/repositories and accession number(s) can be found in the article/**Supplementary Material**.

## Ethics Statement

The studies involving human participants were reviewed and approved by Ethics committees of the National Cancer Institute of Mexico (015/012/ICI, CEI/961/15). The patients/participants provided their written informed consent to participate in this study.

## Author Contributions

Conceptualization, CC-R, CP-P; Experimentation CC-R, AM-G,GC-A; Analysis of results, CC-R, AM-G, E-AP-Y, AC-P, AZ-D, CP-P; Writing-Original Draft Preparation, CC-R, E-AP-Y, AM-G; Writing-Review & Editing, E-AP-Y; CP-P; AC-P, AZ-D Project Administration, CP-P; Funding Acquisition, CP-P. Clinical follow up: JMC. All authors contributed to the article and approved the submitted version

## Conflict of Interest

The authors declare that the research was conducted in the absence of any commercial or financial relationships that could be construed as a potential conflict of interest.

## Publisher’s Note

All claims expressed in this article are solely those of the authors and do not necessarily represent those of their affiliated organizations, or those of the publisher, the editors and the reviewers. Any product that may be evaluated in this article, or claim that may be made by its manufacturer, is not guaranteed or endorsed by the publisher.

## References

[1] Arbyn M Weiderpass E Bruni L Sanjosé S Saraiya M Ferlay J. Estimates of Incidence and Mortality of Cervical Cancer in 2018: A Worldwide Analysis. Lancet Glob Heal (2020) 8:e191–203. doi: 10.1016/S2214-109X(19)30482-6 PMC7025157 31812369

[2] Pilleron S Cabasag CJ Ferlay J Bray F Luciani S Almonte M. Cervical Cancer Burden in Latin America and the Caribbean: Where Are We? Int J Cancer (2020) 147:1638–48. doi: 10.1002/ijc.32956 32150288

[3] Uppal S Gehrig PA Peng K Bixel KL Matsuo K Vetter MH. Recurrence Rates in Patients With Cervical Cancer Treated With Abdominal Versus Minimally Invasive Radical Hysterectomy: A Multi-Institutional Retrospective Review Study. J Clin Onco (2020) 38:1030–40. doi: 10.1200/JCO.19.03012. 32031867

[4] Federico C Sun J Muz B Alhallak K Cosper PF Muhammad N. Localized Delivery of Cisplatin to Cervical Cancer Improves Its Therapeutic Efficacy and Minimizes Its Side Effect Profile. Int J Radiat Oncol Biol Phys (2021) 109:1483–94. doi: 10.1016/J.IJROBP.2020.11.052 PMC8592040 33253820

[5] Costello JF Frühwald MC Smiraglia DJ Rush LJ Robertson GP Gao X. Aberrant CpG-Island Methylation has Non-Random and Tumour-Type–Specific Patterns. Nat Genet (2000) 24(2):132–8. doi: 10.1038/72785 10655057

[6] Fang J Zhang H Jin S. Epigenetics and Cervical Cancer: From Pathogenesis to Therapy. Tumor Biol (2014) 35(6):5083–93. doi: 10.1007/S13277-014-1737-Z 24554414

[7] Filippova M Filippov V Williams VM Zhang K Kokoza A Bashkirova S. Cellular Levels of Oxidative Stress Affect the Response of Cervical Cancer Cells to Chemotherapeutic Agents. BioMed Res Int (2014) 2014:1–14. doi: 10.1155/2014/574659 PMC4248402 25478571

[8] Nahand JS Taghizadeh-boroujeni S Karimzadeh M Borran S Pourhanifeh MH Moghoofei M. microRNAs: New Prognostic, Diagnostic, and Therapeutic Biomarkers in Cervical Cancer. J Cell Physiol (2019) 234:17064–99. doi: 10.1002/JCP.28457 30891784

[9] González-Quintana V Palma-Berré L Campos-Parra AD López−Urrutia E Peralta-Zaragoza O Vazquez-Romo R. MicroRNAs Are Involved in Cervical Cancer Development, Progression, Clinical Outcome and Improvement Treatment Response (Review). Oncol Rep (2016) 35:3–12. doi: 10.3892/OR.2015.4369 26530778

[10] Feng C Dong J Chang W Cui M Xu T. The Progress of Methylation Regulation in Gene Expression of Cervical Cancer. Int J Genomics (2018) 2018:1–11. doi: 10.1155/2018/8260652 PMC5926518 29850477

[11] Lai H-C Lin Y-W Huang THM Yan P Huang R-L Wang H-C. Identification of Novel DNA Methylation Markers in Cervical Cancer. Int J Cancer (2008) 123:161–7. doi: 10.1002/IJC.23519 18398837

[12] Cheung TH Lo KW Yim SF Chan LK Heung MS Chan CS. Epigenetic and Genetic Alternation of PTEN in Cervical Neoplasm. Gynecol Oncol (2004) 93:621–7. doi: 10.1016/J.YGYNO.2004.03.013 15196854

[13] Lee Y Ahn C Han J Choi H Kim J Yim J. The Nuclear RNase III Drosha Initiates microRNA Processing. Nat (2003) 425(6956):415–9. doi: 10.1038/nature01957 14508493

[14] Mitra S Indra DM Bhattacharya N Singh RK Basu PS Mondal RK. RBSP3 Is Frequently Altered in Premalignant Cervical Lesions: Clinical and Prognostic Significance. Genes Chromosom Cancer (2010) 49:155–70. doi: 10.1002/GCC.20726 19885927

[15] Löf-Öhlin ZM Sorbe B Wingren S Nilsson TK. Hypermethylation of Promoter Regions of the APC1A and P16ink4a Genes in Relation to Prognosis and Tumor Characteristics in Cervical Cancer Patients. Int J Oncol (2011) 39:683–8. doi: 10.3892/IJO.2011.1078 21674126

[16] Widschwendter A Gattringer C Ivarsson L Fiegl H Schneitter A Ramoni A. Analysis of Aberrant DNA Methylation and Human Papillomavirus DNA in Cervicovaginal Specimens to Detect Invasive Cervical Cancer and Its Precursors. Clin Cancer Res (2004) 10:3396–400. doi: 10.1158/1078-0432.CCR-03-0143 15161694

[17] Nicole McMillian N Jillian Scavone M Fisher CM Frederick P Gaffney DK George S. Continue NCCN Guidelines Panel Disclosures Ω Gynecologic Oncology Þ Internal Medicine † Medical Oncology § Radiotherapy/Radiation Oncology ≠ Pathology ¥ Patient Advocacy * Discussion Section Writing Committee Emily Wyse ¥ Patient Advocate. J Nat Comprehensive Cancer Net (2019) 17:64–84. doi: 10.6004/JNCCN.2020.0027

[18] Pfister S Schlaeger C Mendrzyk F Wittmann A Benner A Kulozik A. Array-Based Profiling of Reference-Independent Methylation Status (aPRIMES) Identifies Frequent Promoter Methylation and Consecutive Downregulation of ZIC2 in Pediatric Medulloblastoma. Nucleic Acids Res (2007) 35:e51–1. doi: 10.1093/NAR/GKM094 PMC1874664 17344319

[19] Klein CA Schmidt-Kittler O Schardt JA Pantel K Speicher MR Riethmüller G. Comparative Genomic Hybridization, Loss of Heterozygosity, and DNA Sequence Analysis of Single Cells. Proc Natl Acad Sci (1999) 96:4494–9. doi: 10.1073/PNAS.96.8.4494 PMC16360 10200290

[20] Wang J Vasaikar S Shi Z Greer M Zhang B. WebGestalt 2017: A More Comprehensive, Powerful, Flexible and Interactive Gene Set Enrichment Analysis Toolkit. Nucleic Acids Res (2017) 45:W130–7. doi: 10.1093/NAR/GKX356 PMC5570149 28472511

[21] Yu G He Q-Y. ReactomePA: An R/Bioconductor Package for Reactome Pathway Analysis and Visualization. Mol Biosyst (2016) 12:477–9. doi: 10.1039/C5MB00663E 26661513

[22] Kent WJ Sugnet CW Furey TS Roskin KM Pringle TH Zahler AM. The Human Genome Browser at UCSC. Genome Res (2002) 12:996–1006. doi: 10.1101/GR.229102 12045153 PMC186604

[23] Li L-C Dahiya R. MethPrimer: Designing Primers for Methylation PCRs. Bioinformatics (2002) 18:1427–31. doi: 10.1093/bioinformatics/18.11.1427 12424112

[24] Sotlar K Diemer D Dethleffs A Hack Y Stubner A Vollmer N. Detection and Typing of Human Papillomavirus by E6 Nested Multiplex PCR. J Clin Microbiol (2004) 42:3176. doi: 10.1128/JCM.42.7.3176-3184.2004 15243079 PMC446280

[25] Jensen SØ Øgaard N Ørntoft M-BW Rasmussen MH Bramsen JB Kristensen H. Novel DNA Methylation Biomarkers Show High Sensitivity and Specificity for Blood-Based Detection of Colorectal Cancer—a Clinical Biomarker Discovery and Validation Study. Clin Epigenet (2019) 11(1):1–14. doi: 10.1186/S13148-019-0757-3 PMC6854894 31727158

[26] Fukushige S Horii A. DNA Methylation in Cancer: A Gene Silencing Mechanism and the Clinical Potential of Its Biomarkers. Tohoku J Exp Med (2013) 229:173–85. doi: 10.1620/TJEM.229.173 23419314

[27] Gardiner-Garden M Frommer M. CpG Islands in Vertebrate Genomes. J Mol Biol (1987) 196:261–82. doi: 10.1016/0022-2836(87)90689-9 3656447

[28] Naga Ch P Gurram L Chopra S Mahantshetty U. The Management of Locally Advanced Cervical Cancer. Curr Opin Oncol (2018) 30:323–9. doi: 10.1097/CCO.0000000000000471 29994902

[29] Momparler RL. Cancer Epigenetics. Oncogene (2003) 22(42):6479–83. doi: 10.1038/sj.onc.1206774 14528271

[30] Jones PA Baylin SB. The Epigenomics of Cancer. Cell (2007) 128:683–92. doi: 10.1016/J.CELL.2007.01.029 PMC3894624 17320506

[31] Paska AV Hudler P. Aberrant Methylation Patterns in Cancer: A Clinical View. Biochem Med (2015) 25:161–76. doi: 10.11613/BM.2015.017 PMC4470106 26110029

[32] Belinsky SA Nikula KJ Palmisano WA Michels R Saccomanno G Gabrielson E. Aberrant Methylation of P16ink4a Is an Early Event in Lung Cancer and a Potential Biomarker for Early Diagnosis. Proc Natl Acad Sci (1998) 95:11891–6. doi: 10.1073/PNAS.95.20.11891 PMC21736 9751761

[33] Ma J Li R Wang J. Characterization of a Prognostic Four−Gene Methylation Signature Associated With Radiotherapy for Head and Neck Squamous Cell Carcinoma. Mol Med Rep (2019) 20:622–32. doi: 10.3892/MMR.2019.10294 PMC6579992 31180552

[34] Wang Q He Z Chen Y. Comprehensive Analysis Reveals a 4-Gene Signature in Predicting Response to Temozolomide in Low-Grade Glioma Patients. Sage J (2019) 26:1-14. doi: 10.1177/1073274819855118 PMC6558750 31167546

[35] Yang H Zhou L Chen J Su J Shen W Liu B. A Four−Gene Signature for Prognosis in Breast Cancer Patients With Hypermethylated IL15RA. Oncol Lett (2019) 17:4245–54. doi: 10.3892/OL.2019.10137 PMC6447940 30988805

[36] Cardoso M de FS Castelletti CHM de Lima-Filho JL Martins DBG Teixeira JAC. Putative Biomarkers for Cervical Cancer: SNVs, Methylation and Expression Profiles. Mutat Res Mutat Res (2017) 773:161–73. doi: 10.1016/J.MRREV.2017.06.002 28927526

[37] Farkas SA Milutin-Gašperov N Grce M Nilsson TK. Genome-Wide DNA Methylation Assay Reveals Novel Candidate Biomarker Genes in Cervical Cancer. Epigenetics (2013) 8:1213–25. doi: 10.4161/EPI.26346 24030264

[38] Jha AK Nikbakht M Jain V Sehgal A Capalash N Kaur J. Promoter Hypermethylation of P73 and P53 Genes in Cervical Cancer Patients Among North Indian Population. Mol Biol Rep (2012) 39(9):9145–57. doi: 10.1007/S11033-012-1787-5 22729911

[39] Widschwendter A Müller HM Fiegl H Ivarsson L Wiedemair A Müller-Holzner E. DNA Methylation in Serum and Tumors of Cervical Cancer Patients. Clin Cancer Res (2004) 10:565–71. doi: 10.1158/1078-0432.CCR-0825-03 14760078

[40] Filippakopoulos P Picaud S Mangos M Keates T Lambert J-P Barsyte-Lovejoy D. Histone Recognition and Large-Scale Structural Analysis of the Human Bromodomain Family. Cell (2012) 149:214–31. doi: 10.1016/j.cell.2012.02.013 PMC3326523 22464331

[41] Mikeska T Bock C Do H Dobrovic A. DNA Methylation Biomarkers in Cancer: Progress Towards Clinical Implementation. Expert Rev Mol Diagn (2012) 12:473–87. doi: 10.1586/erm.12.45 22702364

[42] Brien GL Remillard D Shi J Hemming ML Chabon J Wynne K. Targeted Degradation of BRD9 Reverses Oncogenic Gene Expression in Synovial Sarcoma. Elife (2018) 7:132–8. doi: 10.7554/ELIFE.41305 PMC6277197 30431433

[43] Dewez M Bauer F Dieu M Raes M Vandenhaute J Hermand D. The Conserved Wobble Uridine tRNA Thiolase Ctu1–Ctu2 Is Required to Maintain Genome Integrity. Proc Natl Acad Sci (2008) 105:5459–64. doi: 10.1073/PNAS.0709404105 PMC2291126 18391219

[44] Delaunay S Rapino F Tharun L Zhou Z Heukamp L Termathe M. Elp3 Links tRNA Modification to IRES-Dependent Translation of LEF1 to Sustain Metastasis in Breast Cancer. J Exp Med (2016) 213:2503–23. doi: 10.1084/JEM.20160397 PMC5068235 27811057

[45] Biswas M Chatterjee SS Boila LD Chakraborty S Banerjee D Sengupta A. MBD3/NuRD Loss Participates With KDM6A Program to Promote DOCK5/8 Expression and Rac GTPase Activation in Human Acute Myeloid Leukemia. FASEB J (2019) 33:5268–86. doi: 10.1096/FJ.201801035R 30668141

[46] Bahrami A Hasanzadeh M Hassanian SM ShahidSales S Ghayour-Mobarhan M Ferns GA. The Potential Value of the PI3K/Akt/mTOR Signaling Pathway for Assessing Prognosis in Cervical Cancer and as a Target for Therapy. J Cell Biochem (2017) 118:4163–9. doi: 10.1002/JCB.26118 28475243

[47] Deng J Bai X Feng X Ni J Beretov J Graham P. Inhibition of PI3K/Akt/mTOR Signaling Pathway Alleviates Ovarian Cancer Chemoresistance Through Reversing Epithelial-Mesenchymal Transition and Decreasing Cancer Stem Cell Marker Expression. BMC Cancer (2019) 19(1):1–12. doi: 10.1186/S12885-019-5824-9 31234823 PMC6591840

[48] Dong C Wu J Chen Y Nie J Chen C. Activation of PI3K/AKT/mTOR Pathway Causes Drug Resistance in Breast Cancer. Front Pharmacol (2021) 118:143. doi: 10.3389/FPHAR.2021.628690 PMC8005514 33790792

