# Supplementary material for: Gene Promoter-Methylation Signature as Biomarker to Predict Cisplatin-Radiotherapy Sensitivity in Locally Advanced Cervical Cancer
Source: Front Oncol. 2022 Mar 3;12:773438. doi: 10.3389/fonc.2022.773438 (PMC8963763; doi:10.3389/fonc.2022.773438)
Supplement: Supplementary file 1 [file DataSheet_1.pdf]

Supplementary Material

Supplementary Figure 1

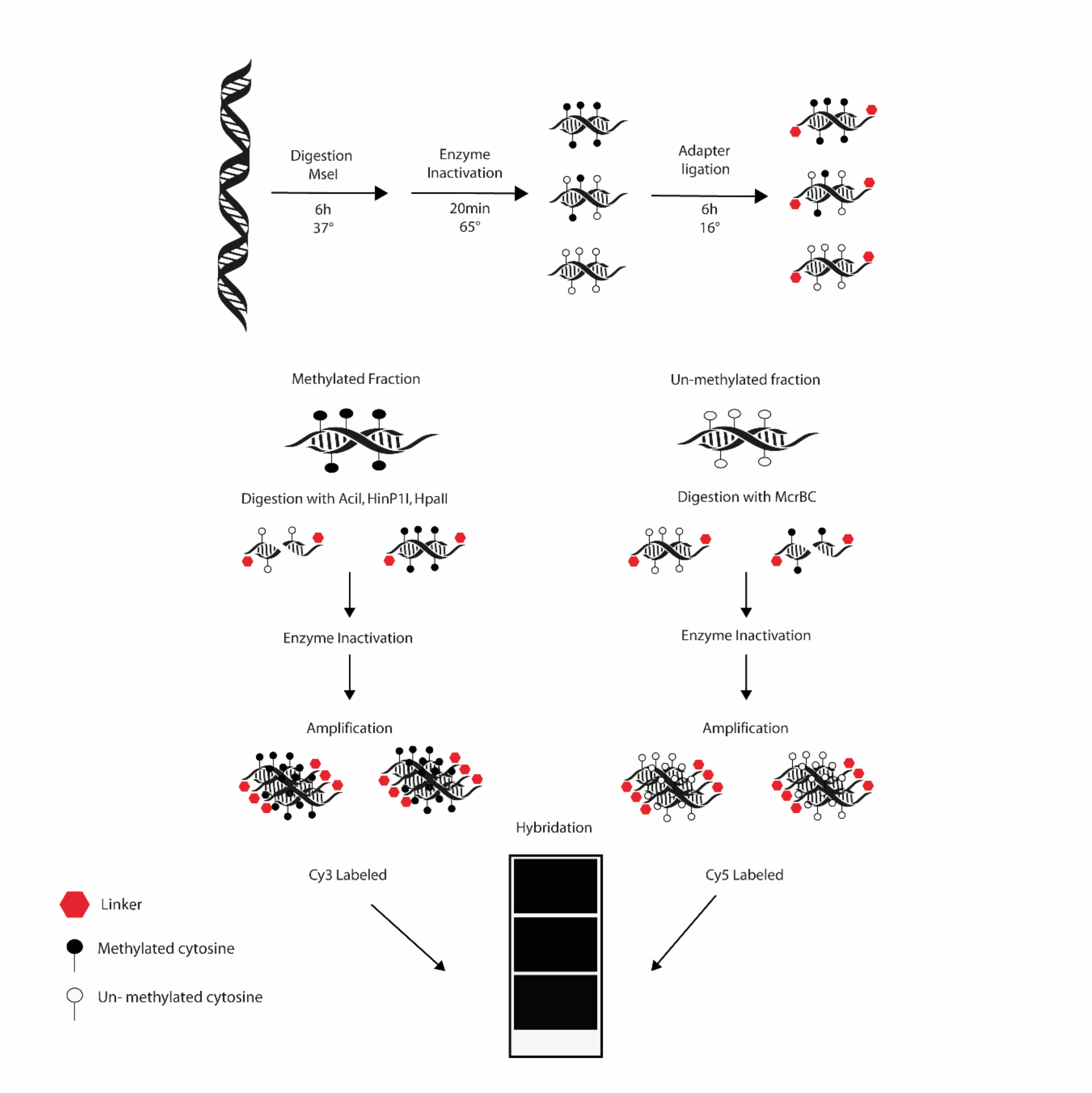

Supplementary Figure 1. Flowchart and method performance to aPRIMES assay

Supplementary Figure 2

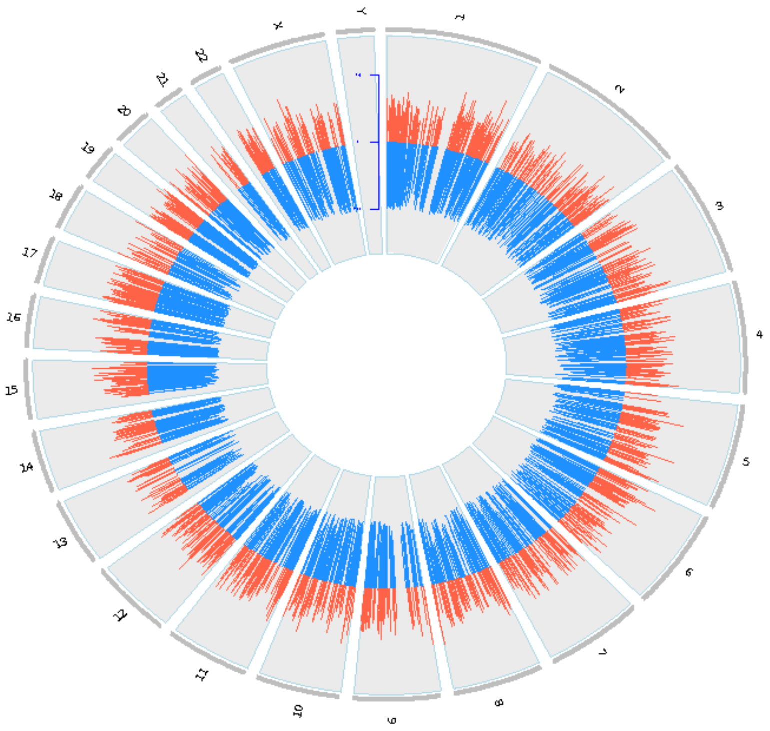

Supplementary Figure 2. Circos plot representing the differentially methylated regions across the genome in cervical cancer patients. The blue inner ring represents the bi-weight of the significative methylated regions, meanwhile red outer ring shows hypermethylated chromosome areas.

Supplementary Figure 3

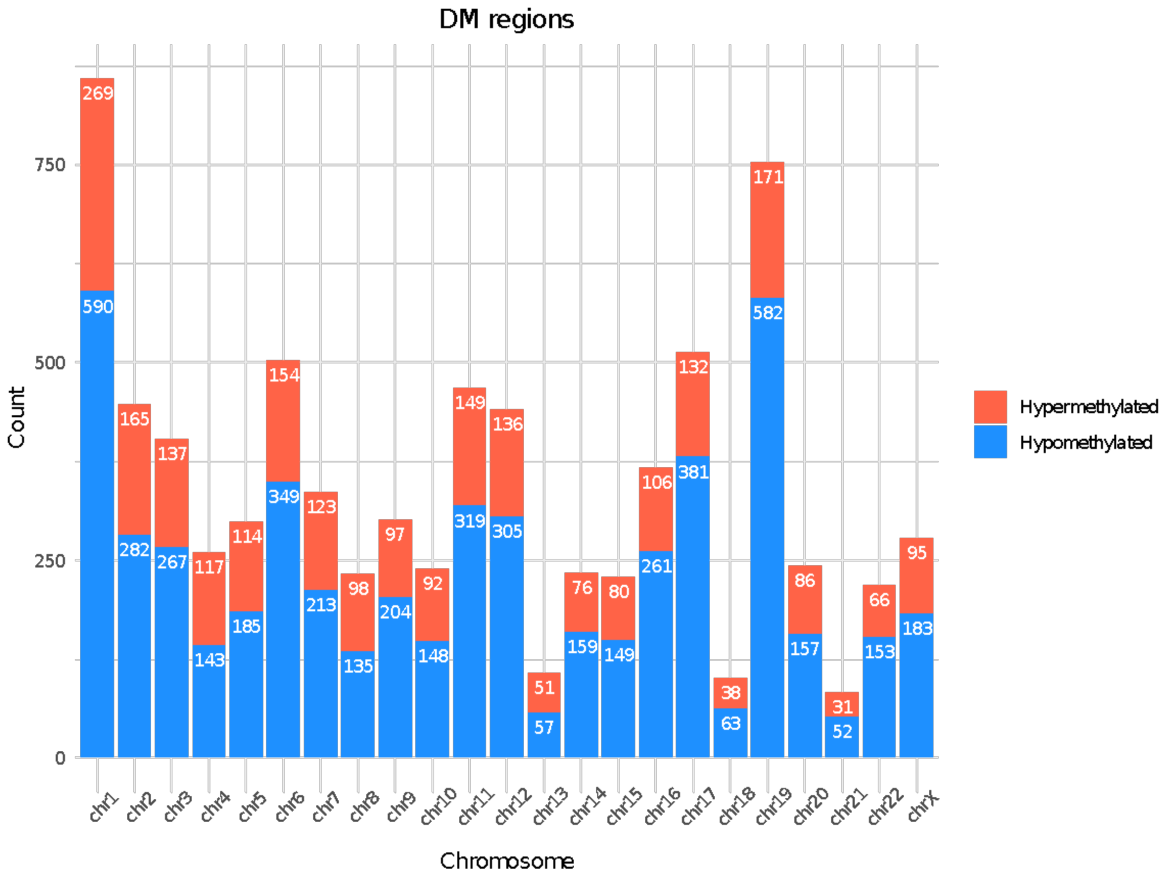

Supplementary Figure 3. Number of regions differentially methylated across the genome. The barplot represents the number of regions differentially methylated per chromosome where light blue shows hypermethylated regions and dark blue hypomethylated.

Supplementary Table 1. MSP primers.

Gene		Methylated	Un-methylated
**BRD9**	Fw	GAATTTTTTATTAGGAACGTAAGCG	GAATTTTTTATTAGGAATGTAAGTG
	Rv	CTACGAAAATAAAACGACGACG	TCCTACAAAAATAAAACAACAACAC
**CTU1**	Fw	CGAGTAGTTGGGATTATAGGTTTAC	TTTTGAGTAGTTGGGATTATAGGTTTAT
	Rv	AAAAAATCTAAAAAAAACGCGAA	ATCAAAAAATCTAAAAAAAACACAAA
**DOCK8**	Fw	AGTTTAGCGTTGGTGTGAAGC	GATAGTTTAGTGTTGGTGTGAAGTG
	Rv	GAAACGAACGAAATTATTCGAAA	ACAAAACAAACAAAATTATTCAAAA
